# Amplitude Versus Angle (AVA) feature restoration in prestack gathers via dictionary learning

**DOI:** 10.1371/journal.pone.0343701

**Published:** 2026-03-20

**Authors:** Yang Gao, Xuewen Shi, Dongjun Zhang, Chang Wang, Ruhua Zhang, Yanwen Feng

**Affiliations:** Research Institute of Shale Gas, PetroChina Southwest Oil & Gas Field Company, Chengdu, Sichuan, China; Wadia Institute of Himalayan Geology, INDIA

## Abstract

With the expansion of oil and gas exploration into deep and complex reservoirs, the prestack amplitude versus angle (AVA) inversion technique faces challenges due to amplitude attenuation and phase distortion caused by formation absorption effects, which limit the accuracy of seismic attribute characterization. To address the limitations of existing compensation methods, particularly poor noise robustness and insufficient lateral continuity, we propose a dictionary learning–based AVA feature restoration method for prestack gathers. First, local AVA features extracted from well–log data are used to construct a training dataset using a sliding time window, and the K–Singular Value Decomposition (K–SVD) algorithm is used to train an overcomplete dictionary that sparsely represents attenuation-free signals. Subsequently, the dictionary learning process is embedded into the absorption compensation objective function, where dictionary atoms and sparse coefficients are alternately optimized via orthogonal matching pursuit (OMP) algorithm and gradient descent (GD) algorithm to achieve effective signal-noise separation. Synthetic tests show that, compared with conventional methods, the proposed approach restores weak reflection energy, compensates for angle-dependent amplitude distortion, and exhibits reduced dependence on Q-model accuracy with markedly improved noise robustness. Field data applications demonstrate the advantages of the proposed method in improving lateral continuity and restoring AVA responses under complex geological conditions, providing data-driven support for high-precision prestack elastic-parameter inversion.

## 1 Introduction

With the global expansion of oil and gas exploration into deep and complex reservoirs, exploration targets have gradually shifted from conventional hydrocarbon reservoirs with clear structural features to subtle lithologic reservoirs controlled by the coupling of lithology, physical properties, and fluid parameters [1]. Such reservoirs typically exhibit strong heterogeneity, thin interbedded layers, and abrupt spatial variations in physical properties, rendering traditional seismic interpretation methods based on structural identification inadequate due to insufficient resolution and weakened fluid responses. In this context, prestack amplitude versus angle (AVA) inversion has emerged as a core technique for shale gas sweet spot prediction and tight sandstone gas reservoir classification [2]. However, the viscoelastic nature of subsurface media distorts the AVA characteristics of seismic waves, significantly limiting the accuracy of seismic attributes in hydrocarbon characterization. Therefore, developing stratigraphic absorption compensation techniques for prestack gathers to reconstruct high-fidelity AVA responses is critical for improving exploration accuracy.

The absorption properties of subsurface media are quantitatively characterized by the quality factor Q [3]. Based on Q dispersion theory, multiple attenuation models have been established [4,5], laying the theoretical foundation for absorption compensation. Current compensation methods fall into two categories: Q-compensated reverse time migration (Q-RTM) [6] and inverse Q filtering [7]. While Q-RTM enhances imaging resolution and amplitude fidelity for deep thin reservoirs by compensating for stratigraphic absorption, its performance is sensitive to Q-model errors and noise interference [8], with high computational costs limiting its widespread application. In contrast, inverse Q filtering is generally more computationally efficient and cost-effective. Robinson [9] proposed a phase-correction compensation method that effectively mitigates phase dispersion; however, it does not restore the amplitudes of reflection events. Hargreaves [10] enhanced the efficiency of phase correction by implementing the compensation algorithm in the frequency domain using the fast Fourier transform (FFT) and provided a theoretical framework based on wave-field extrapolation. Wang [11] proposed the stability-factor method, rewriting the original amplitude-compensation function in fractional form and introducing a stability factor to prevent the amplification of high-frequency noise. Subsequent refinements improved the robustness of wave-field-extrapolation-based inverse Q filtering [12]. Zhang [13] pioneered the formulation of absorption attenuation as a least-squares inversion problem within a Bayesian framework, regularizing the solution with an $L_{2}$-norm term. Compared with wave-field-extrapolation methods, this approach yielded notably more robust compensation results. Wang [14] introduced compressed sensing theory and reformulated the minimization problem using the difference of convex functions algorithm, which decomposes the original problem into two subproblems. Ma [15] proposed multitrace absorption compensation by integrating lateral predictability and reflection structure constraints with $L_{1}$-norm regularization, achieving high lateral continuity and signal-to-noise ratio. Wang [16] introduced structural tensor regularization into an $L_{1-2}$ norm inversion framework, further enhancing stability and spatial continuity.

Poststack absorption compensation is relatively mature; however, these methods cannot be directly applied to prestack data because they neglect the coupled effects of raypath propagation and absorption. prestack seismic signals exhibit nonstationary attenuation, with dynamic attenuation variations across propagation paths distorting AVA responses and degrading elastic parameter inversion accuracy [17]. To address these challenges, recent studies have concentrated on developing advanced prestack compensation techniques. [18] first proposed offset-dependent absorption compensation, establishing a theoretical framework. Li et al. (2014) [19] decomposed absorption into depth- and offset-dependent components, mitigating amplitude versus offset (AVO) distortion and improving vertical resolution. Zhang [20] corrected the AVO response in the time–frequency– space domain by imposing low-frequency constraints derived from the attenuation differences between high- and low-frequency components in prestack gathers, thereby achieving a high-fidelity restoration of the AVO signature. Aharchaou [21] performed local-angle decomposition using the τ−p transform and applied Q compensation to prestack data, thereby attenuating incoherent noise and spatial aliasing. However, because this approach assumes a constant-Q model and cannot accommodate spatiotemporal variations in Q, it is subject to significant limitations. Cheng [22] combined Tikhonov regularization with an $L_{1}$-norm constraint to enhance the stability of angle-gather compensation. However, these methods primarily rely on mathematical manipulations of seismic data and insufficiently incorporate inherent geological information, often inducing spatial distortions of AVA response features in structurally complex areas.

To address these limitations, we propose a dictionary learning-based prestack AVA feature recovery method that achieves stable compensation of prestack gathers within a data‑driven inversion framework. First, a forward model of nonstationary viscoelastic media is established to explicitly characterize angle‑dependent amplitude attenuation and phase distortion. Next, local AVA signatures are extracted from well‑log–derived gathers via a sliding time window to construct the training dataset [23]. Subsequently, the K‑singular value decomposition (K-SVD) algorithm [24] is employed to learn an overcomplete dictionary capable of sparsely representing attenuation-free signals. Finally, the dictionary learning process is integrated into the absorption compensation objective function, with dictionary atoms and sparse coefficients alternately optimized using the orthogonal matching pursuit (OMP) algorithm [25] and gradient descent (GD) algorithm [26]. Through this compensation framework, the AVA features can be recovered more robustly while considering the formation characteristics.

## 2 Theory

### 2.1 Nonstationary seismic data forward modeling

In real subsurface viscoelastic media, the phase velocity can be written as [27]:


$$\frac{1}{v(\omega )}=\frac{1}{v_{r}}\left[1-\frac{1}{\pi Q_{r}}\ln\left(\frac{\omega }{\omega_{b}}\right)\right]\approx \frac{1}{v_{r}}{\left|\frac{\omega }{\omega_{b}}\right|}^{-\gamma }$$ (1)

Where *ω* is the angular frequency; $v_{r}$ and $Q_{r}$ are the velocity and quality factor at the reference frequency; and $\gamma =1/(\pi Q_{r})$. In practice, $\omega_{h}$ is typically set to the maximum frequency within the seismic bandwidth.

The complex wavenumber of seismic waves is defined as [28]:


$$k(\omega )=\frac{\omega }{v(\omega )}-i\alpha (\omega )$$ (2)

where α(ω) is the attenuation coefficient, given by:


$$\alpha (\omega )=\frac{\left|w\right|}{2v_{r}Q_{r}}$$ (3)

Seismic records at a specific depth can be modeled as the product of reflection coefficients and harmonic waves [29]:


$$w(\omega )e^{i(\omega t-k(\omega )z)}r(z)$$ (4)

where w(ω) is the seismic wavelet spectrum, and r(z) denotes the reflection coefficient.

**Fig 1:**
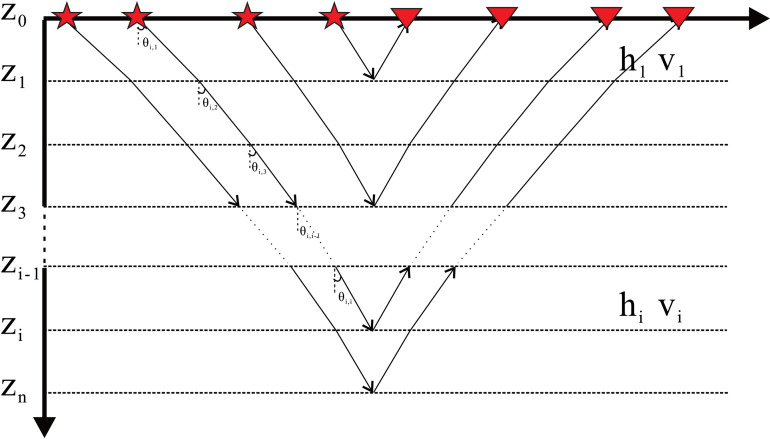
Propagation paths of incidence angle gathers in a horizontally layered medium model.


For a horizontally layered medium model (Fig 1), the propagation of seismic waves is described as follows. Let the thickness between the *i*th and (i−1)th layers be $h_{i}$, the layer velocity be $v_{i}$ and the incidence angle be $\theta_{i}$. If a seismic wave propagates from the $Z_{0}$ layer to the $Z_{i}$ layer and is recorded by a surface receiver, the process can be formulated as:

$$s(Z_{i},\omega ,t,\theta )=r(Z_{i},\theta )w(\omega )e^{i\omega t}e^{-i\omega \sum_{k=1}^{i}\frac{2h_{k}}{v(Z_{i})\cos\theta_{i,k}}}$$ (5)

where $\theta_{i,k}$ is the incidence angle at the $Z_{k}$ layer, determined by Snell’s law.

Substituting the phase velocity from Eq (1) and replacing the propagation distance with travel time, Eq (5) becomes


$$s(Z_{i},\omega ,t,\theta )=r(Z_{i},\theta )w(\omega )e^{i\omega t}e^{-i\omega \sum_{k=1}^{i}\frac{\tau_{k}}{\cos\theta_{i,k}}{\left|\frac{\omega }{\omega_{h}}\right|}^{-\gamma }}e^{-\omega \sum_{k=1}^{i}\frac{\tau }{2\cos\theta_{i,k}Q_{r}}{\left|\frac{\omega }{\omega_{h}}\right|}^{-\gamma }}$$ (6)

Summing harmonic waves generated by reflection coefficients $r(Z_{i},\theta )$ at different depths yields the complete seismic record:


$$s(\omega ,t,\theta )=\sum_{k=1}^{i}r(Z_{k},\theta )w(\omega )e^{i\omega t}e^{-i\omega \sum_{k=1}^{i}\frac{\tau_{k}}{\cos\theta_{i,k}}{\left|\frac{\omega }{\omega_{h}}\right|}^{-\gamma }}e^{-\omega \sum_{k=1}^{i}\frac{\tau }{2\cos\theta_{i,k}Q_{r}}{\left|\frac{\omega }{\omega_{h}}\right|}^{-\gamma }}$$ (7)

Summing over all angular frequencies gives the time-domain forward model for prestack data in attenuating media:


$$s(t,\theta )=\int_{-\infty }^{+\infty }\sum_{k=1}^{i}r(z_{k},\theta )w(\omega )e^{i\omega t}e^{-i\omega \sum_{k=1}^{i}\frac{\tau_{k}}{\cos\theta_{i,k}}{\left|\frac{\omega }{\omega_{h}}\right|}^{-\gamma }}e^{-\omega \sum_{k=1}^{i}\frac{\tau }{2\cos\theta_{i,k}Q_{r}}{\left|\frac{\omega }{\omega_{h}}\right|}^{-\gamma }}d\omega$$ (8)

**Fig 2:**
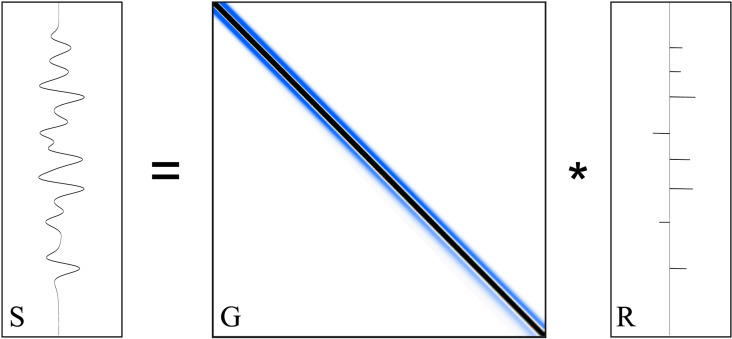
The forward modeling workflow. **G** represents the nonstationary wavelet matrix, **R** is the reflectivity series, and **S** is the nonstationary seismic trace.

The matrix-vector form of Eq (8) is:


$$\begin{array}{c} {}[\begin{array}{c} s(t_{1},\theta )\\ s(t_{2},\theta )\\ \vdots \\ s(t_{N,\theta }) \end{array}]=[\begin{array}{cccc} g(t_{1},\tau_{1},\theta ) & g(t_{1},\tau_{2},\theta ) & \cdots & g(t_{1},\tau_{N},\theta )\\ g(t_{2},\tau_{1},\theta ) & g(t_{2},\tau_{2},\theta ) & \cdots & g(t_{2},\tau_{N},\theta )\\ \vdots & \vdots & \ddots & \vdots \\ g(t_{N},\tau_{1},\theta ) & g(t_{N},\tau_{2},\theta ) & \cdots & g(t_{N},\tau_{N},\theta ) \end{array}][\begin{array}{c} r(\tau_{1},\theta )\\ r(\tau_{2},\theta )\\ \vdots \\ r(\tau_{N,\theta }) \end{array}]\; \end{array}$$ (9)

which can be abbreviated as:


𝐒=GR (10)

where **S** and **R** represent the seismic data and reflection coefficients, respectively, and **G** is the nonstationary wavelet matrix (Fig 2).

### 2.2 Inversion framework based on dictionary learning

In geophysical inversion, the objective is to estimate the model parameter vector **R** from the observed data vector **S** and the forward operator **G**. However, due to the inherently ill-posed nature of this inverse problem, Eq (9) is typically solved through regularized least-squares formulations, which can be expressed as:


$$𝐑=\arg\min_{𝐑}{\left\|𝐒-𝐆𝐑\right\|}_{2}^{2}+\lambda f(𝐑)$$ (11)

where f(𝐑) denotes the regularization term and *λ* controls the trade-off between data fidelity and model constraints. When using the $L_{1}$-norm constraint, Eq (11) can be written as


$$𝐑=\arg\min_{𝐑}{\left\|𝐒-𝐆𝐑\right\|}_{2}^{2}+\lambda {\left\|𝐑\right\|}_{1}$$ (12)

**Fig 3:**
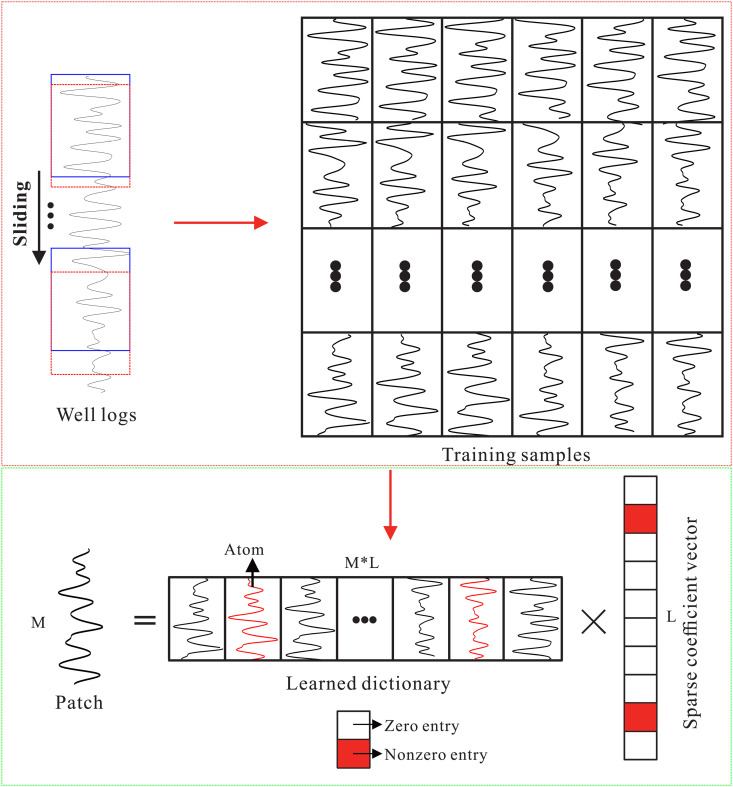
Dictionary learning and sparse coding.

While Total Variation (TV) and Tikhonov (TK) regularization remain widely adopted in geophysical inversion [30], these methods often overlook geological response characteristics embedded in seismic data. To enhance adaptability to complex geological structures, we introduce a dictionary learning-constrained inversion framework [23], where sparse representations of geological features are explicitly incorporated as prior knowledge. Based on the spatial correlation inherent in seismic data, we postulate the following assumptions: within a designated spatial range, each seismic data patch can be effectively represented by a globally defined sparse dictionary, and the sparse dictionary derived from selected logging data can serve as a close approximation to this global dictionary. The process of dictionary learning can be described as the following energy minimizing problem:


$$\begin{array}{c} \Theta =\arg\min_{𝐃,𝐀}{\left\|ℜ(𝐗)-𝐃𝐀\right\|}_{F}^{2}\\ \text{subject to}\;\forall i:{\left\|{𝐚}_{i}\right\|}_{0}\leq K\;(K\ll L) \end{array}$$ (13)

where $ℜ(𝐗)=\left[{𝐱}_{1},{𝐱}_{2},\ldots ,{𝐱}_{N}\right]\in {\mathbb{R}}^{M\times N}$ denotes a training set consisting of *N* small image blocks, each of length *M*. $𝐃\in {\mathbb{R}}^{M\times L}$ represents a dictionary matrix, and $𝐀=\left[{𝐚}_{1},{𝐚}_{2},\ldots ,{𝐚}_{N}\right]\in {\mathbb{R}}^{L\times N}$ denotes a coefficient matrix composed of *N* sets of sparse coefficients. ${\left\|{𝐚}_{i}\right\|}_{0}$ indicates the number of nonzero elements in the sparse vector $a_{i}$, *K* specifies the sparsity level (i.e., the maximum number of nonzero coefficients), and *L* represents the number of atoms in the dictionary. The Eq (13) can be solved by the K-SVD algorithm [24]. Fig 3 shows a diagram of dictionary learning and sparse coding.

Accordingly, the objective function for the absorption compensation inversion based on dictionary learning is defined as follows:


$$\begin{array}{c} \Psi =\min_{𝐑,\{{𝐚}_{i}\},𝐃}\frac{1}{2}{\left\|𝐒-𝐆𝐑\right\|}_{2}^{2}+\lambda \sum_{i=1}^{N}{\left\|{ℜ}_{i}(𝐑)-𝐃{𝐚}_{i}\right\|}_{2}^{2}\\ \text{subject to}\;{\left\|{𝐚}_{i}\right\|}_{0}\leq K,\;i=1,2,\ldots ,N \end{array}$$ (14)

where **S** denotes the observed data, **G** is the nonstationary wavelet matrix, and **R** represents the data to be recovered. The operator ${ℜ}_{i}()$ extracts the i-th local patch from **R**. **D** is the global dictionary derived from log data, $a_{i}$ is the sparse representation of the i-th local patch under **D**, and *λ* is the regularization parameter. In order to solve the complicated Eq (14), we employ an alternating iterative optimization scheme. The original problem is addressed by sequentially solving the following subproblems:

Step 1: The initial input vector ${𝐑}^{\left(0\right)}$ is obtained by solving a least-squares problem:


$$𝐉({𝐑}^{\left(0\right)})=\min\frac{1}{2}{\left\|𝐒-𝐆𝐑\right\|}_{2}^{2}+\mu {\left\|𝐑\right\|}_{2}^{2}$$ (15)

Step 2: For each local block, after fixing the current estimate and dictionary, solve the following equation:


$$\begin{array}{c} {𝐚}_{i}^{\left(k+1\right)}=\arg\min_{{𝐚}_{i}}{\left\|{ℜ}_{i}({𝐑}^{\left(k\right)})-𝐃{𝐚}_{i}\right\|}_{2}^{2}\\ \text{subject to}\;{\left\|{𝐚}_{i}\right\|}_{0}\leq K \end{array}$$ (16)

The orthogonal matching pursuit (OMP) algorithm [25] can be efficiently employed to solve this problem, yielding the sparse representation coefficients for each data block.

Step 3: Fix all sparse coefficients and update **R**:


$${𝐑}^{\left(k+1\right)}=\arg\min_{𝐑}\frac{1}{2}{\left\|𝐒-𝐆{𝐑}^{\left(k\right)}\right\|}_{2}^{2}+\lambda \sum_{i=1}^{N}{\left\|{ℜ}_{i}({𝐑}^{\left(k\right)})-𝐃{𝐚}_{i}^{\left(k+1\right)}\right\|}_{2}^{2}$$ (17)

Since ${ℜ}_{i}()$ typically extracts overlapping local blocks, block averaging is considered during reconstruction. The gradient descent method [26] can be applied for optimization and updating.

Step 4: Iteration and convergence criteria. Repeat steps 2 and 3 until the convergence condition is satisfied:


$$\frac{{\left\|{𝐑}^{\left(k+1\right)}-{𝐑}^{\left(k\right)}\right\|}_{F}}{{\left\|{𝐑}^{\left(k\right)}\right\|}_{F}}<\varepsilon$$ (18)

where *ε* is the preset tolerance threshold.

This alternating iterative optimization strategy leverages the complementary advantages of the data fidelity term and the sparse representation constraint in dictionary learning. It not only ensures the accuracy of the fitted observation data but also effectively suppresses noise and preserves spatial continuity through local sparse representation. The solution procedure and parameter-tuning strategy are summarized in the pseudocode below.

**Pseudocode: Dictionary-learning–constrained absorption compensation**.

**Input:** attenuated gather *S*; per-angle operator *G*; patch extractor $\left\{{ℛ}_{i}\right\}$; patch size *M*, strides *sp*; dictionary size *L*; sparsity *K*; weight *λ*.

**Output:** compensated result *R*.

**1) Patch extraction and training. Extract patches with overlap determined by *M* and *sp*; Train the dictionary** $D\;\in \;{\mathbb{R}}^{M\times L}$ **using K-SVD for** $n_{iter}=20$**; and** $L_{2}$**-normalize atoms after each update.**

**2) Initialization. Compute a ridge/Tikhonov initial estimate** $R^{\left(0\right)}$**. Set the step size** $\tau \in \;[10^{-3},10^{-1}]\;\backslash )$**, tolerance** $=5\times 10^{-4}$**, and the maximum number of outer alternations** =100.

**3) Alternating optimization.**

*(a) Sparse coding (OMP):* for each patch *i*, select atoms by maximum absolute correlation; stop when the residual <10^–6^ or the iteration count reaches the maximum; obtain $a_{i}^{\left(k+1\right)}$.

*(b) Gradient step on R:* compute $g^{\left(k\right)}=G^{⊤}(GR^{\left(k\right)}-S)+\lambda \sum_{i}{ℛ}_{i}^{⊤}({ℛ}_{i}(R^{\left(k\right)})-Da_{i}^{\left(k+1\right)}$ and update $R^{\left(k+1\right)}=R^{\left(k\right)}-\tau \;g^{\left(k\right)}$; fuse overlaps by block averaging.

*(c) Stopping:* if $\|R^{\left(k+1\right)}-R^{\left(k\right)}{\|}_{2}/\|R^{\left(k\right)}{\|}_{2}<5\times 10^{-4}$ or the outer count reaches 100, terminate.

**Return** $R^{\left(k+1\right)}$.

*Notes:* Given the presence of multiple key hyperparameters, a one-factor-at-a-time (controlled-variable) tuning strategy is recommended, using RMSE as the evaluation metric; the optimal configuration is then determined via repeated parameter sweeps.

## 3 Numerical experiments

### 3.1 Synthetic data

#### 3.1.1 Noise resistance and stability analysis.

**Fig 4:**
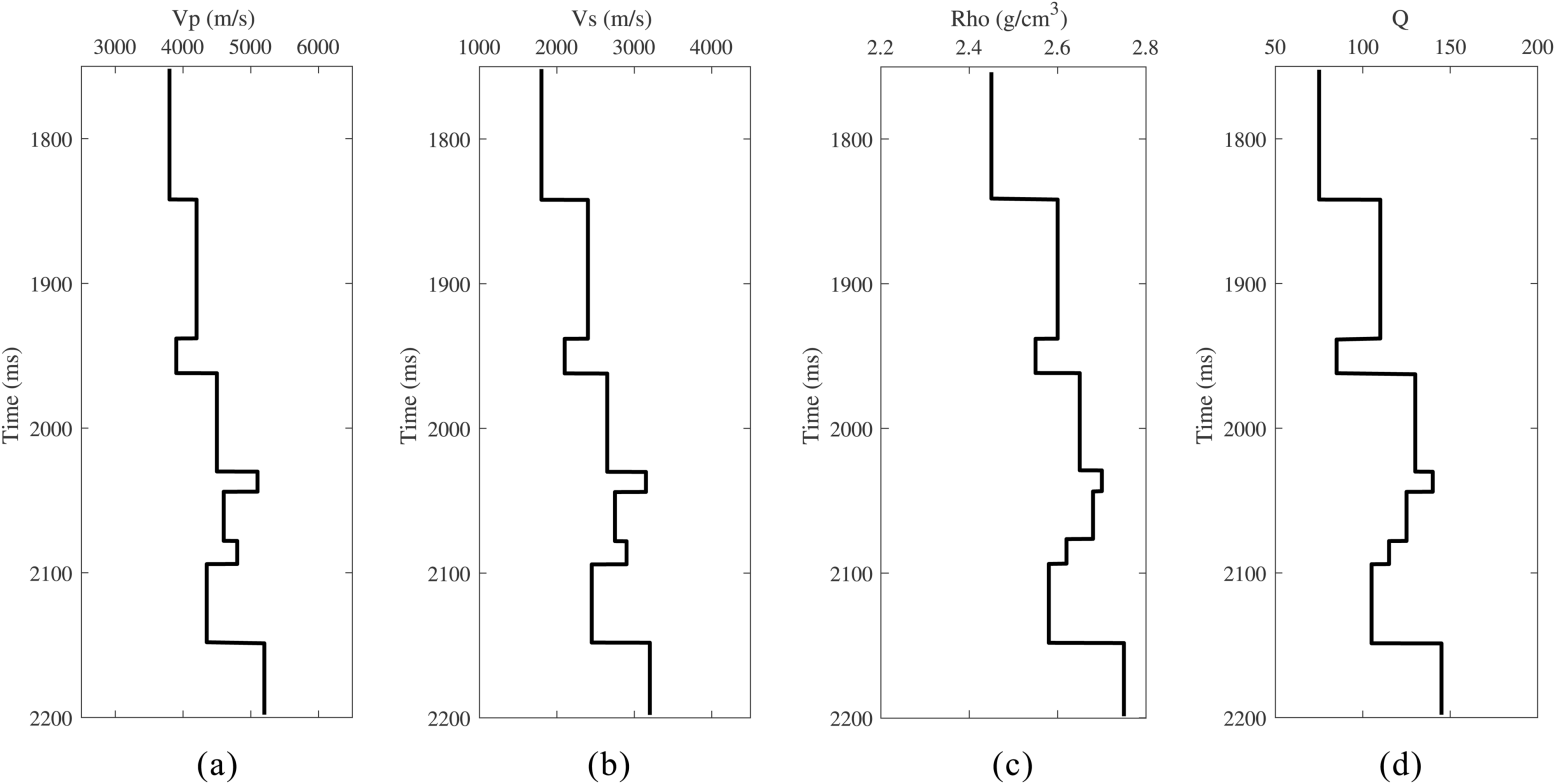
Parameters of the horizontally layered medium model. **(a)** P-wave velocity. **(b)** S-wave velocity. **(c)** Density. **(d)** Quality factor.

**Fig 5:**
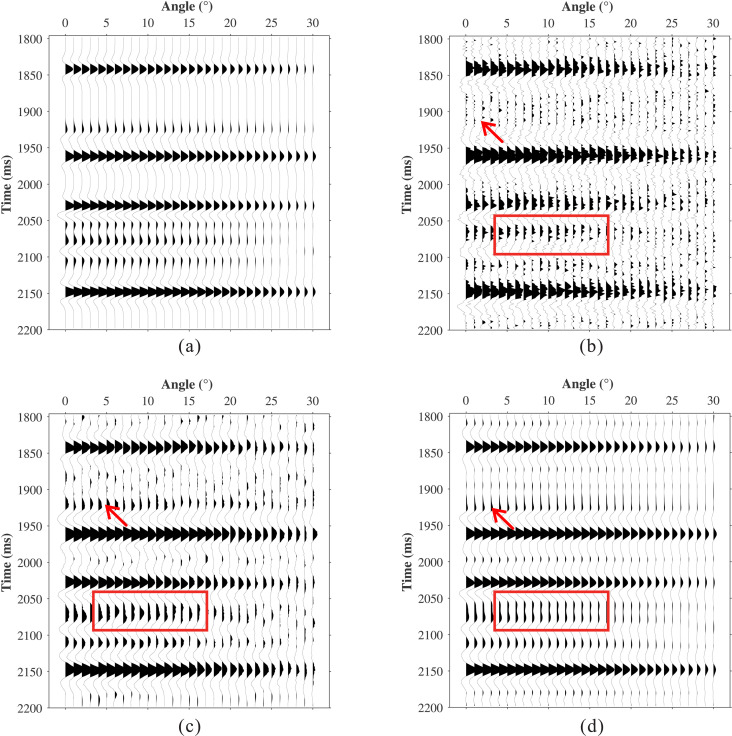
Comparison of synthetic data compensation results. **(a)** Stationary data. **(b)** Attenuated data. **(c)** L1-norm. **(d)** The Proposed method.

To systematically validate the effectiveness of the proposed method, a nine-layer horizontally layered medium model was designed. The model parameters include P-wave velocity $V_{p}$, S-wave velocity $V_{s}$, density Rho, and quality factor *Q* (Fig 4). A Ricker wavelet with a dominant frequency of 30 Hz was used as the source. Under idealized conditions (ignoring geometric spreading, transmission loss, and stretch effects during normal moveout), reflection coefficients for incidence angles ranging from 0° to 40° (in 1° increments) were calculated using the Aki-Richards approximation. prestack angle gathers were synthesized using Eq (9), with the stationary data shown in Fig 5(a). The data after absorption attenuation are displayed in Fig 5(b), where 20% random noise was added to test the robustness of the proposed method. Fig 5(b) shows that absorption attenuation both reduces the reflection energy of all horizons and narrows the seismic bandwidth, thereby distorting the original amplitude response. For example, the two weak events at 2060 ms are merged into a single seismic event, while the weak event near 1920 ms is completely suppressed (red arrow).

The conventional $L_{1}$-norm constrained method and the proposed method were applied to the attenuated data in Fig 5(b), with results shown in Fig 5(c) and 5(d), respectively. Under these conditions, the $L_{1}$-norm method achieves its best performance with *λ* = 0.001, whereas the proposed method is configured with a window size *M* = 26, dictionary size *L* = 78, sparsity level *K* = 8, and regularization parameter *λ* = 0.05. The conventional method achieved an RMSE of 0.0339, whereas the proposed method achieved 0.0105. Both methods effectively compensate for amplitude attenuation and recover weak reflection events. However, the conventional method exhibits limited noise immunity and produces discontinuous events, and it cannot eliminate the waveform interference within the red-boxed area. In contrast, the proposed method attenuates noise while recovering signal energy, preserving amplitude features with lateral continuity. This is because the proposed method not only accounts for absorption differences at different angles but also incorporates geological characteristics and spatial correlations by introducing dictionary learning, effectively achieving the separation of signal and noise.

**Fig 6:**
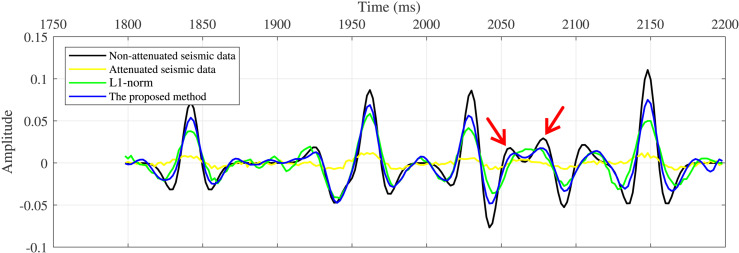
Single-trace comparison of compensation results.

**Fig 7:**
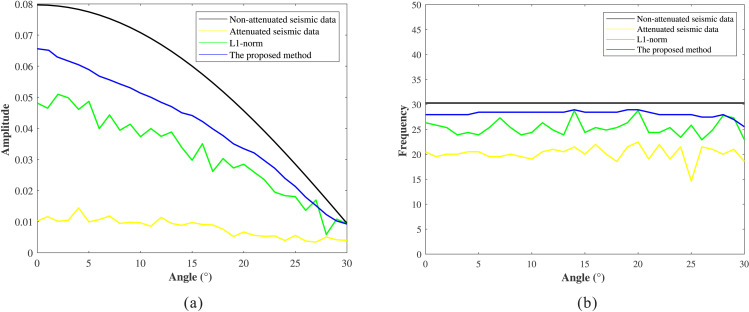
Analysis of compensation results. **(a)** AVA curves. **(b)** PFVA curves.

A single trace at a 9 incidence angle was extracted for detailed comparison (Fig 6). The proposed method (blue curve) closely matches the waveform characteristics of the stationary data (black curve) and successfully recovers weak reflections (red arrows), whereas the conventional method fails to restore weak amplitudes adequately. To further analyze amplitude and frequency recovery, we extracted amplitude versus angle (AVA) curves (Fig 7(a)) and peak-frequency versus angle (PFVA) curves (Fig 7(b)) for the first reflection event. Results indicate that angle-dependent absorption attenuation causes significant distortion of the AVA response (yellow curve), resulting in substantial deviation from the theoretical trend (black curve). Both methods recover the AVA trend. However, the AVA curve produced by the conventional method exhibits noise-induced oscillations, whereas the proposed method yields a smoother, geologically consistent trend that closely follows the theoretical response. The PFVA curves further indicate that absorption causes the peak frequency to decrease with increasing angle (yellow curve). While both the conventional and proposed methods enhance the peak frequency, the proposed method achieves superior performance at larger angles (blue curve).

#### 3.1.2 Q value sensitivity analysis.

**Fig 8:**
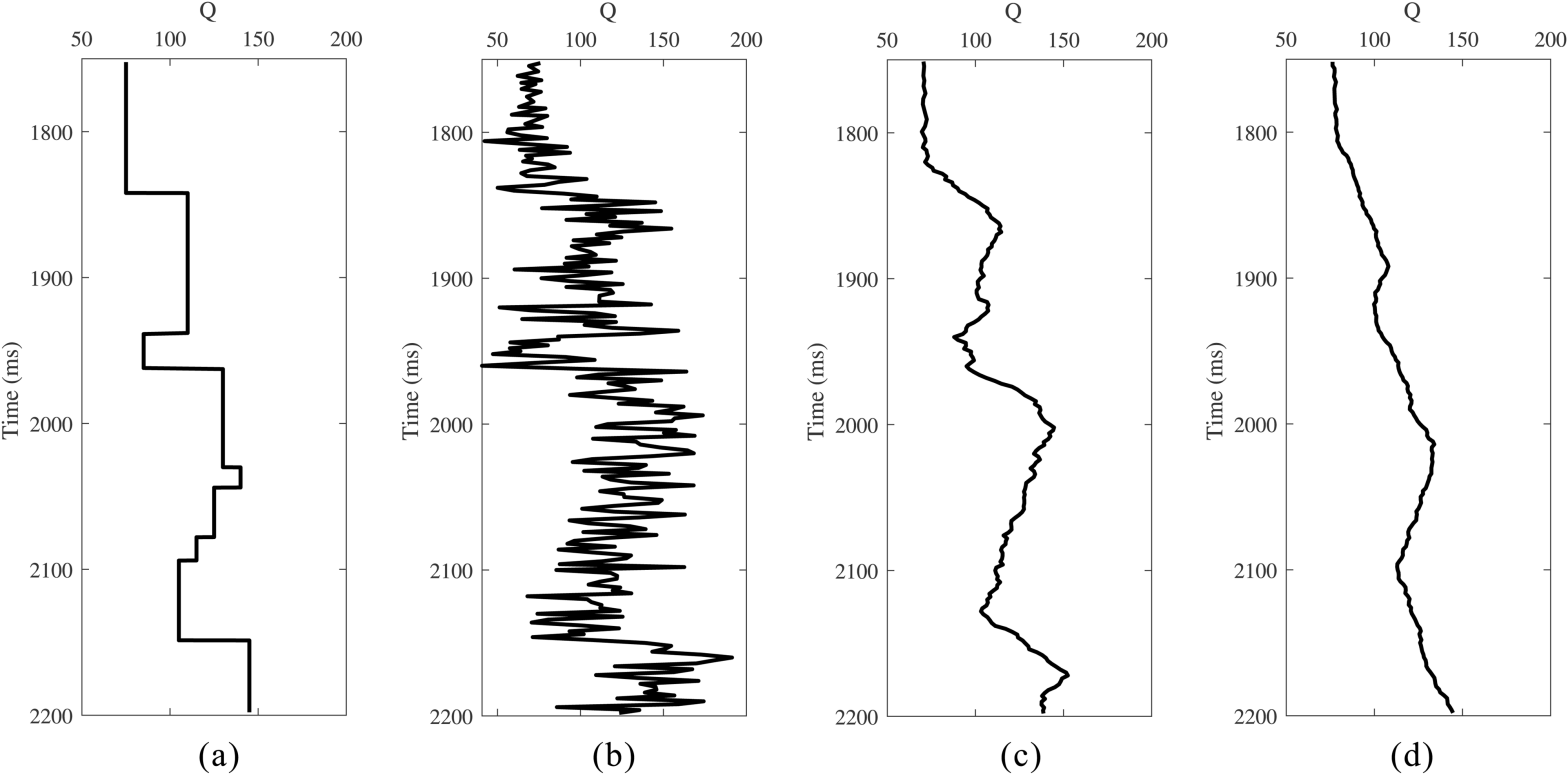
Q models. **(a)** Initial Q model. **(b)** Q models with 20% Gaussian random errors. (c) 31-point smoothing. (d) 51-point smoothing.

**Fig 9:**
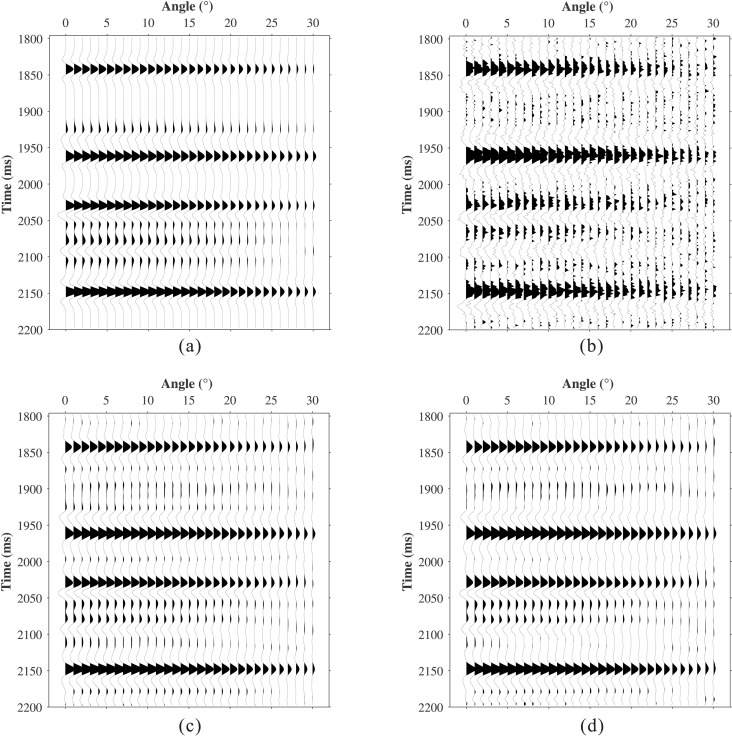
Compensation results under different Q models. **(a)** Stationary data. **(b)** Attenuated data. (c) 31-point smoothing. (d) 51-point smoothing.

In the process of prestack seismic data absorption attenuation compensation, the accuracy of the Q value model directly affects the compensation performance. However, in actual production, it is often difficult to establish a high-precision Q value model. To systematically evaluate the dependence of the proposed method on Q-model precision, a sensitivity analysis was conducted: (1) Add 20% random perturbation on the basis of the reference Q model shown in Fig 8(a); (2) The perturbation Q model (Fig 8(b)) was smoothed at 31 points (Fig 8(c)) and 51 points (Fig 8(a)) respectively to generate the low-frequency Q model; (3) The proposed method was applied to the attenuated gathers (Fig 9(b)) using these low-frequency Q models.

**Fig 10:**
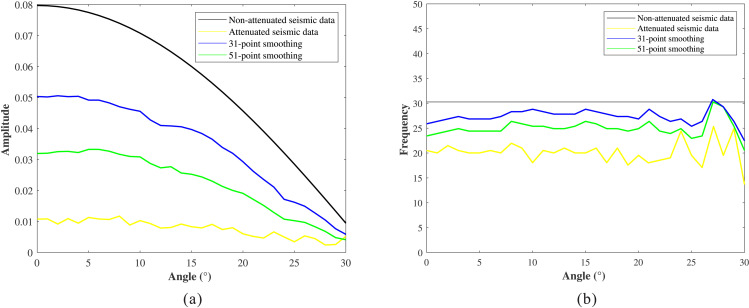
Analysis of compensation results in Fig 9. **(a)** AVA curves. **(b)** PFVA curves.

Fig 9(c) and 9(d) show the compensation results obtained using 31-point and 51-point low-frequency background Q models, respectively. In both cases, the amplitude energy and phase distortions in the original gathers are effectively corrected, and interference effects are mitigated. To further evaluate the compensation performance, AVA and PFVA curves corresponding to the seismic event near 2150 ms are extracted and compared in Fig 10(a) and 10(b), respectively. The results indicate that, even when using low-frequency Q models, the compensated gathers exhibit AVA and PFVA trends that remain highly consistent with the theoretical trends, demonstrating the proposed method’s low sensitivity to the accuracy of the Q model.

#### 3.1.3 Key parameters for dictionary learning.

**Fig 11:**
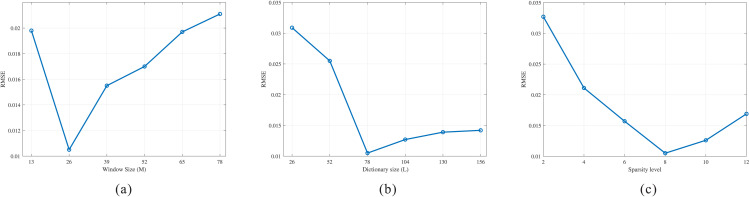
Sensitivity of RMSE to patch window size, dictionary size, and sparsity level. **(a)** Variation of Root Mean Square Error (RMSE) with Window Size. **(b)** Variation of Root Mean Square Error (RMSE) with Dictionary Size. **(c)** Variation of Root Mean Square Error (RMSE) with Sparsity.

To assess the impact of dictionary-learning hyperparameters, we conducted controlled experiments. Using a one-factor-at-a-time design, we examined how patch length, dictionary size, and sparsity level affect the compensation performance. The experimental settings are summarized in Table 1, and the results are presented in Fig 11.

**Table 1:** Sensitivity of RMSE to patch window size *M*, dictionary size *L*, and sparsity level *K.*

A. Window size *M* (fixed $\begin{array}{c} L=3*M\; \end{array}$, $\begin{array}{c} K=8\; \end{array}$)						
*M* (samples)	13	26	39	52	65	78
RMSE	0.0198	**0.0105**	0.0155	0.0170	0.0197	0.0211
**B. Dictionary size *L*** (fixed $\begin{array}{c} M=26\; \end{array}$, $\begin{array}{c} K=8\; \end{array}$)						
*L* (atoms)	1*M	2*M	3*M	4*M	5*M	6*M
RMSE	0.0309	0.0255	**0.0105**	0.0127	0.0139	0.0142
**C. Sparsity level *K*** (fixed $\begin{array}{c} M=26\; \end{array}$, $\begin{array}{c} L=78\; \end{array}$)						
*K* (atoms)	2	4	6	8	10	12
RMSE	0.0327	0.0211	0.0157	**0.0105**	0.0126	0.0169

With the dictionary size fixed at L=3M and sparsity at K=8, the root mean square error (RMSE) exhibits a clear U-shaped dependence on the window length. Small windows lack sufficient context, hindering the separation of absorption effects from noise and increasing bias. Very large windows violate local stationarity, thereby increasing variance. With the window fixed at M=26 and sparsity at K=8, the RMSE first decreases and then increases with the dictionary size. When L≤2M, the dictionary is under-expressive and fails to represent angle-dependent structure. When L≥4M, redundant atoms promote noise adherence and worsen conditioning. With the window and dictionary fixed at M=26 and L=78(3M, the RMSE is also U-shaped. Low *K* underfits structural components and leaves compensation residuals, whereas high *K* overfits noise and weakens denoising. Overall, a robust default configuration is M=26, L=3M=78, K=8, where all three sweeps attain the same global minimum (RMSE=0.0105). The regularization parameter *λ* was determined by a sweep over $\left[10^{-3},10^{-1}\right]$ using RMSE as the evaluation metric; the optimal value in this study is λ=0.05.

### 3.2 Field data

**Fig 12:**
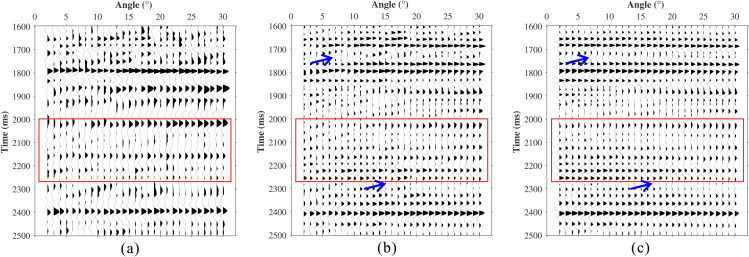
Comparison of field data compensation results. **(a)** Raw data. **(b)** Conventional compensation. **(c)** Proposed method.

To validate the applicability of the proposed method under complex geological conditions, field angle gathers from a certain area in southern China were selected for analysis. The prestack angle gathers from the study area are shown in Fig 12(a). Due to absorption attenuation, the amplitude energy is weakened, particularly within the 2000–2270 ms window. This weakening causes event discontinuities and prevents reliable AVA identification. Both a conventional absorption‑compensation method and the proposed method were applied to these data. The result of the conventional method is displayed in Fig 12(b), and the result of the proposed method in Fig 12(c). Both approaches effectively restore deep‑layer amplitude energy and correct phase distortion. However, relative to the conventional scheme, the proposed method yields markedly improved spatial continuity (blue arrows).

**Fig 13:**
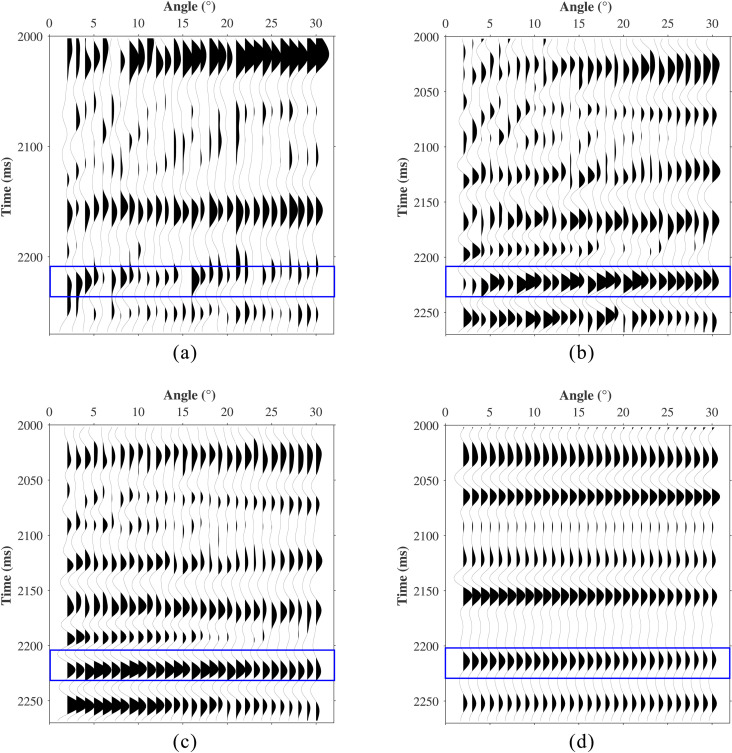
Local Zoomed-In View. **(a)** Raw data. **(b)** Conventional compensation. **(c)** Proposed method. **(d)** Well-forward synthetic record.

**Fig 14:**
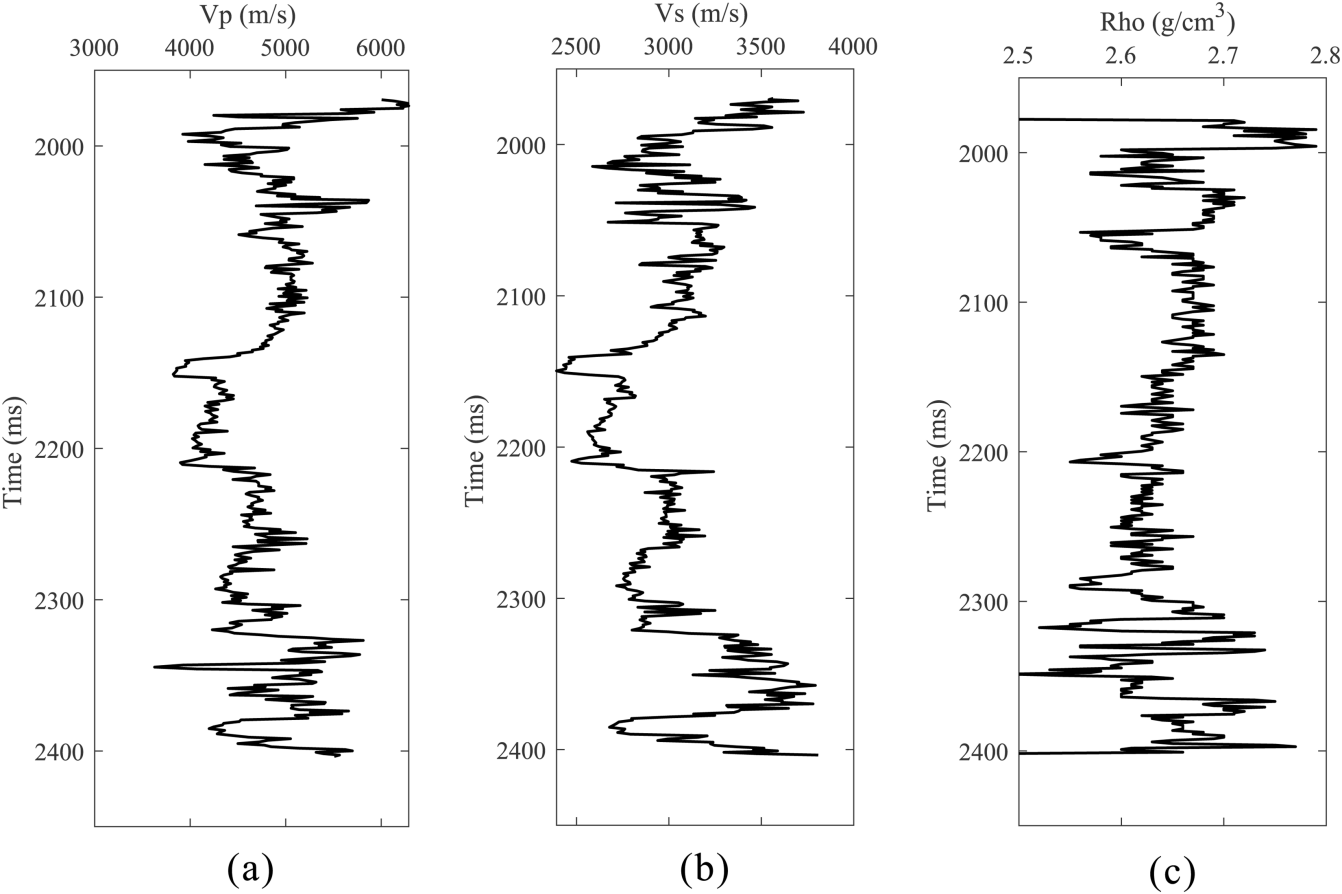
Actual logging data. **(a)** P-wave velocity. **(b)** S-wave velocity. **(c)** Density.

Fig 13 presents an enlarged view of the red‑boxed region in Fig 12. Fig 13(d) shows the angle gather synthetically generated from well logs. Well information (velocity and density) is plotted in Fig 14. It can be seen that the proposed scheme (Fig 13(c)) both preserves spatial continuity and correctly recovers the AVA signature (blue rectangle), matching the synthetics more closely.

**Fig 15:**
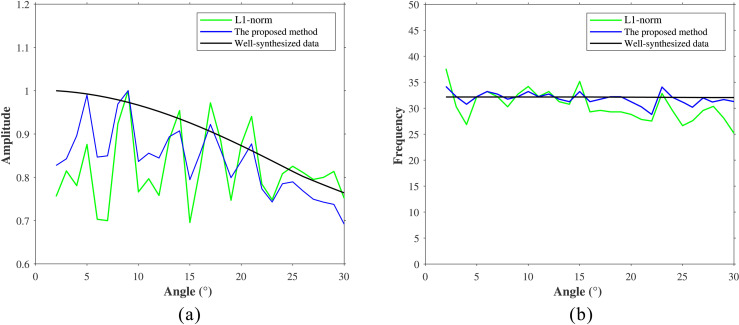
Analysis of field data Compensation Results. **(a)** AVA curves. **(b)** PFVA curves.

The AVA and PFVA curves extracted from the key events within the blue rectangle are compared in Fig 15. In both panels the black curve is the well‑synthetic reference, the blue curve is the result of the proposed method, and the green curve is from the conventional method. The AVA curves (Fig 15(a)) demonstrate that the proposed method more accurately recovers both amplitude energy and trend relative to the well synthetics. The PFVA curves (Fig 15(b)) show that only the proposed method successfully restores large‑angle signal energy, whereas the conventional method fails to do so.

## 4 Conclusion

This study addresses amplitude versus angle (AVA) distortion caused by absorption attenuation in prestack seismic data by proposing a dictionary learning-based single-channel absorption compensation method. A nonstationary forward model is constructed to explicitly characterize spatially varying absorption effects, while a sparse dictionary trained on well-log data integrates prior geological knowledge into the inversion framework, enabling synergistic optimization of sparse signal representation and noise suppression. Synthetic tests confirm the method’s capability to recover weak reflection energy in deep layers, compensate for angle-dependent amplitude distortion, and maintain stability even under Q-model perturbations of 20%. Field data applications show that compensated gathers align closely with well-derived synthetics, and AVA curves match theoretical predictions, verifying the method’s reliability. Compared to conventional single-channel approaches, this method enables selective compensation of signals and noise through dictionary learning-based method, offering a novel solution for high-resolution seismic exploration in complex reservoirs.
